# Hematic auto-management and extraction for arterial line (HAMEL), a blood-preserving arterial line system: an animal study

**DOI:** 10.1038/s41598-023-33539-8

**Published:** 2023-04-26

**Authors:** Hanyoung Lee, Jae-myeong Lee

**Affiliations:** Division of Acute Care Surgery, Department of Surgery, Korea University Anam Hospital, Korea University Medical Center, Goryeodae-ro 73, Seongbuk-gu, Seoul, 02841 Republic of Korea

**Keywords:** Mechanical engineering, Laboratory techniques and procedures

## Abstract

Invasive arterial line insertion is a common procedure in the intensive care unit ICU; however, it can cause unnecessary blood loss while procuring blood for laboratory tests. To reduce blood loss resulting from flushing out the arterial line dead space, we developed a new blood-preserving arterial line system: Hematic Auto-Management & Extraction for arterial Line (HAMEL, MUNE Corp.). Five male three-way crossbred pigs were used to evaluate the necessary amount of blood to be withdrawn before sampling to produce accurate results. We then tested whether the traditional sampling method and the HAMEL system showed non-inferior results for blood tests. Blood gas (CG4 + cartridge) and chemistry (CHEM8 + cartridge) analyses were used for comparison. The total unnecessary blood loss in the traditional sampling group was 5 mL/sample. For HAMEL, withdrawing 3 mL of blood before sampling yielded hematocrit and hemoglobin results within 90% confidence interval of traditional sampling group. Most intra-class correlation coefficients between the traditional sampling and HAMEL system groups were > 0.90. When compared to the traditional sampling method, withdrawal of 3 mL with HAMEL was sufficient before blood sampling. Utilization of the HAMEL system was not inferior to the traditional hand-sampling method. In addition, no unnecessary blood loss occurred in the HAMEL system.

## Introduction

The intensive care unit (ICU) is one of the busiest places in hospitals where many bedside procedures are performed. Invasive arterial line (A-line) insertion is a common procedure performed on patients in the ICU for real-time blood pressure monitoring and ease of arterial blood sampling.

Unfortunately, approximately 4–10 mL of blood should be discarded before actual blood sampling to remove fluids from the dead space of the A-line^1–3^, which can cause unnecessary blood loss each time the blood is sampled. Empirically, majority of South Korean physicians are discarding 5 mL of blood in practice. The majority of patients in the ICU develop anemia for various reasons^4–7^ and frequently require transfusion; however, blood transfusions can also lead to complications. Hence, it is important to prevent unnecessary blood loss^8,9^.

To reduce blood loss associated with flushing out the A-line dead space, it has been suggested that fluids in the A-line dead space should be recirculated rather than discarded^10^. As an intervention to prevent unnecessary blood loss as well as reduce exterior environmental exposure time, we present a new blood-preserving A-line system—Hematic Auto-Management and Extraction for arterial-Line (HAMEL).

To test the device and simulate its relevance to human biology, we developed a porcine model to evaluate (1) how much blood should be withdrawn prior to sampling for accurate blood test results, and (2) if the test results using the blood sample obtained by the device are consistent with those obtained by the traditional hand sampling method. We expected that a clearing volume of 3 mL, which is double the dead-space volume of 1.5 mL, would be the smallest clearing volume needed to yield results in agreement with the control group. We predicted no significant difference between test results of experiment and control group.

## Methods

According to the study design, the estimated amount of blood loss per one experiment was at least 115 mL (For first sub-experiment; 45 mL, 11 blood samples, for second sub-experiment; 70 mL, 20 blood samples) plus 30 mL (for additional blood sampling due to the test errors). Estimated time spent for all procedures (sedation and intubation of pig, first and second sub-experiment) was at least 3 h. We could afford only one experiment per one pig for this reason, and as we planned to obtain 50 blood samples for each of first and second sub-experiment, we used a total of five pigs for this study. No inclusion or exclusion criteria were set. No randomization or blinding was performed. Our study strictly followed recommendations in the ARRIVE guidelines. The specimens were male, three-way crossbred pigs and conventional animals. As the characteristics of each pig were similar and the study details of each experiment were identical, there was no need to consider potential confounders. The mean age of the pigs was 98.4 ± 2.7 days (range 95–102 days), and the mean body weight was 47.8 ± 1.8 kg (range 47.0–50.2 kg). For proper A-line insertion and management, the pigs were maintained under general anesthesia with endotracheal intubation and ventilation. Zoletil^®^ (tiletamine/zolazepam, 2 mmol/kg) was administered intramuscularly to induce sedation, followed by inhalational anesthesia with isoflurane (dose range: 0.5–2 L/min). There were no humane endpoints due to general anesthesia. Room temperature was maintained at 20–21 °C, and the body temperature of the pigs was maintained at 37 °C using a warming device. Fluid was supplied by intravenous administration of 500 mL Hartmann dextrose solution.

An A-line was inserted into both femoral arteries using the cut-down method and linked to a vital sign monitor to observe blood pressure and heart rate. The left side was used as the traditional sampling group (control group), and the right side was used as the HAMEL sampling group (experimental group). Same A-line catheter was used for both sides. The A-line catheter was 20 gauge in diameter and 5 cm in length (Arrow^®^, Teleflex Incorporated, Pennsylvania, United States). The average length from the A-line distal connecting site to the stopcock port was 27.94 ± 0.635 cm (TruWave™ 3 cc/60 in Edwards Lifesciences, California, United States). The dead-space volume in the A-line catheter was 0.5 mL, and the volume from the distal end of the A-line catheter to the blood sampling site was 1 mL; therefore, the total estimated dead space volume was 1.5 mL.

We used i-STAT1® (Abbott Inc.) for the blood gas (CG4 + cartridge) and chemistry (CHEM8 + cartridge) analyses. The required blood sample volume for the device was 1 mL.

**Figure 1:**
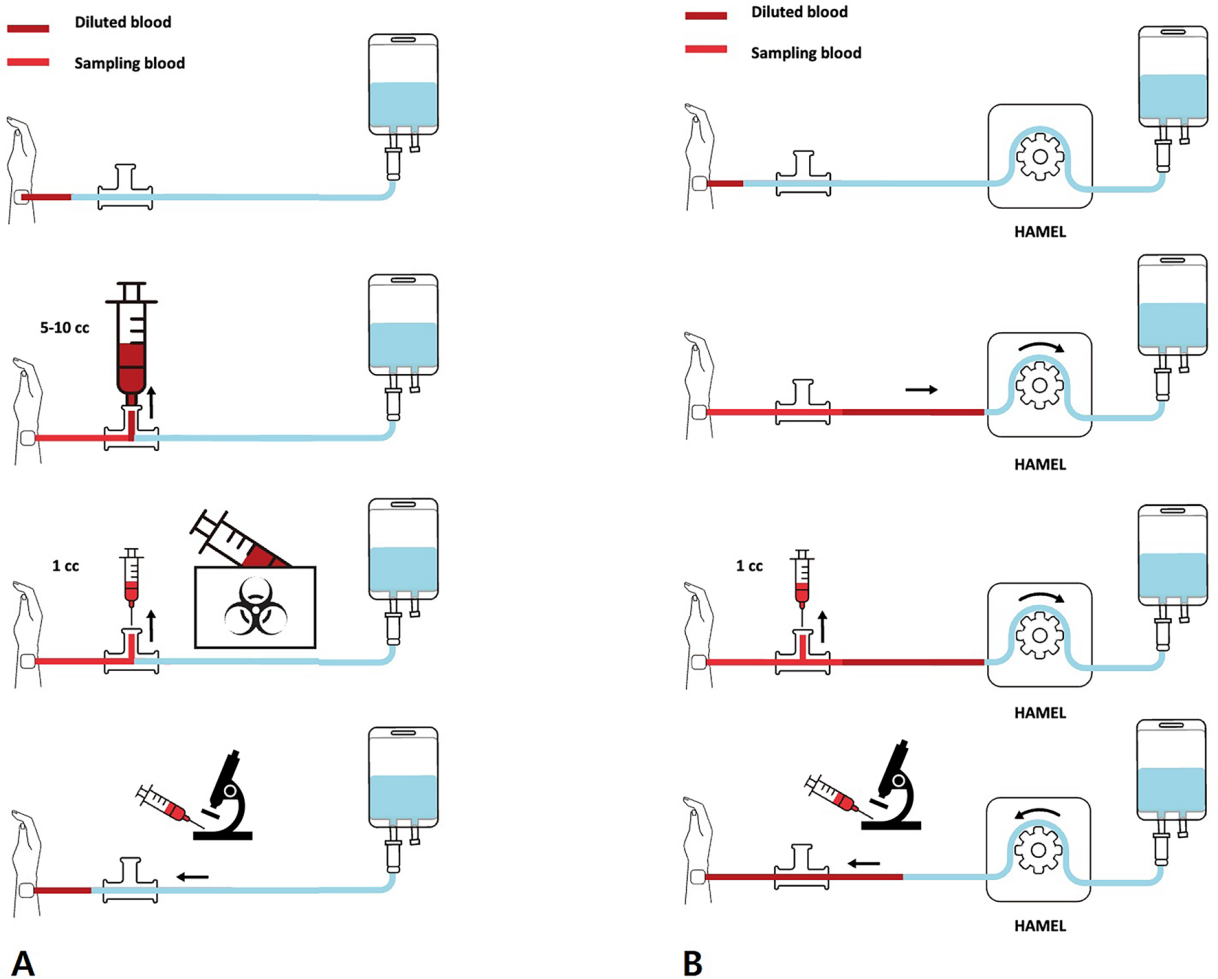
Comparison of HAMEL mechanism to traditional blood sampling method. HAMEL is automated for clearing out and flushing in of normal saline-mixed blood. Since entire process is performed inside closed system of fluid line, there is no unnecessary blood loss.

The HAMEL device was operated using a peristaltic pump, and a button was used to invert the flow direction. The distal side was linked to a normal saline pressure bag, and the proximal side was connected to the A-line. Dead space clearing and normal saline flushing were automated with a simple button; only the actual blood sampling process was performed manually (Fig. 1). For clearing dead space, initial volume of 5 mL was regurged through fluid line. After blood sampling was done, HAMEL device flushed 8 mL of normal saline through fluid line, which can return regurged blood to the patient without unnecessary blood loss.

The first sub-experiment evaluated the amount of blood to be cleared prior to blood sampling for producing exact blood test results. An A-line lumen is typically filled with fluids, such as normal saline, prior to use; hence, we aimed to determine the degree to which samples were diluted by testing hemoglobin and hematocrit levels. Initially, after clearing 5 mL of blood, we obtained 1 mL blood samples from the control group. Serial sampling of the experimental group was performed via the A-line with prior clearing volumes of 0.5, 1, 1.5, 2, 2.5, 3, 3.5, 4, 4.5, and 5 mL. After each blood sample was taken from the experimental group, 5 mL of normal saline was infused through the A-line to flush the dead space, and we waited 5 min before the next sample to prevent resampling of the diluted blood. By repeating these procedures in five pigs, we obtained five samples for each clearing volume.

The second sub-experiment tested whether the traditional sampling method and the HAMEL system showed statistically similar blood test results. The A-lines for both the control and experimental groups were monitored to determine any significant differences in blood pressure. For the control group, 5 mL of blood was cleared prior to sampling 1 mL of blood. After sampling, 5 mL of normal saline was flushed through the A-line. In the experimental group, as mentioned above, 5 mL was regurgitated, and after sampling 1 mL of blood, 8 mL of normal saline was flushed through the A-line.

We chose to flush 8 mL due to the results of first sub-experiment. When we flushed 5 mL of normal saline, there were signs of remnant blood inside fluid line lumen. The fluid line was flushed clearly with flushing volume more than 8 mL.

Both samples were taken simultaneously with 10 serial samples at 5 min intervals. By repeating these procedures for five pigs, we obtained 50 samples for each of the control and experimental groups.

SPSS 25 (IBM Inc.) was used for statistical analysis. A Bland–Altman plot was used to analyze the adequate clearing volume range. A 90% confidence interval was used to decrease the differences between the control and experimental groups.

### Ethics approval and consent to participate

The study protocol was reviewed and approved by the Institutional Review Board of the Korea University School of Medicine Institutional Animal Care and Use Committee (approval no. KOREA-2021-0203).

All procedures were conducted in accordance with the revised Animals (Scientific Procedures) Act 1986 in the UK and were performed according to the relevant guidelines.

## Results

**Figure 2:**
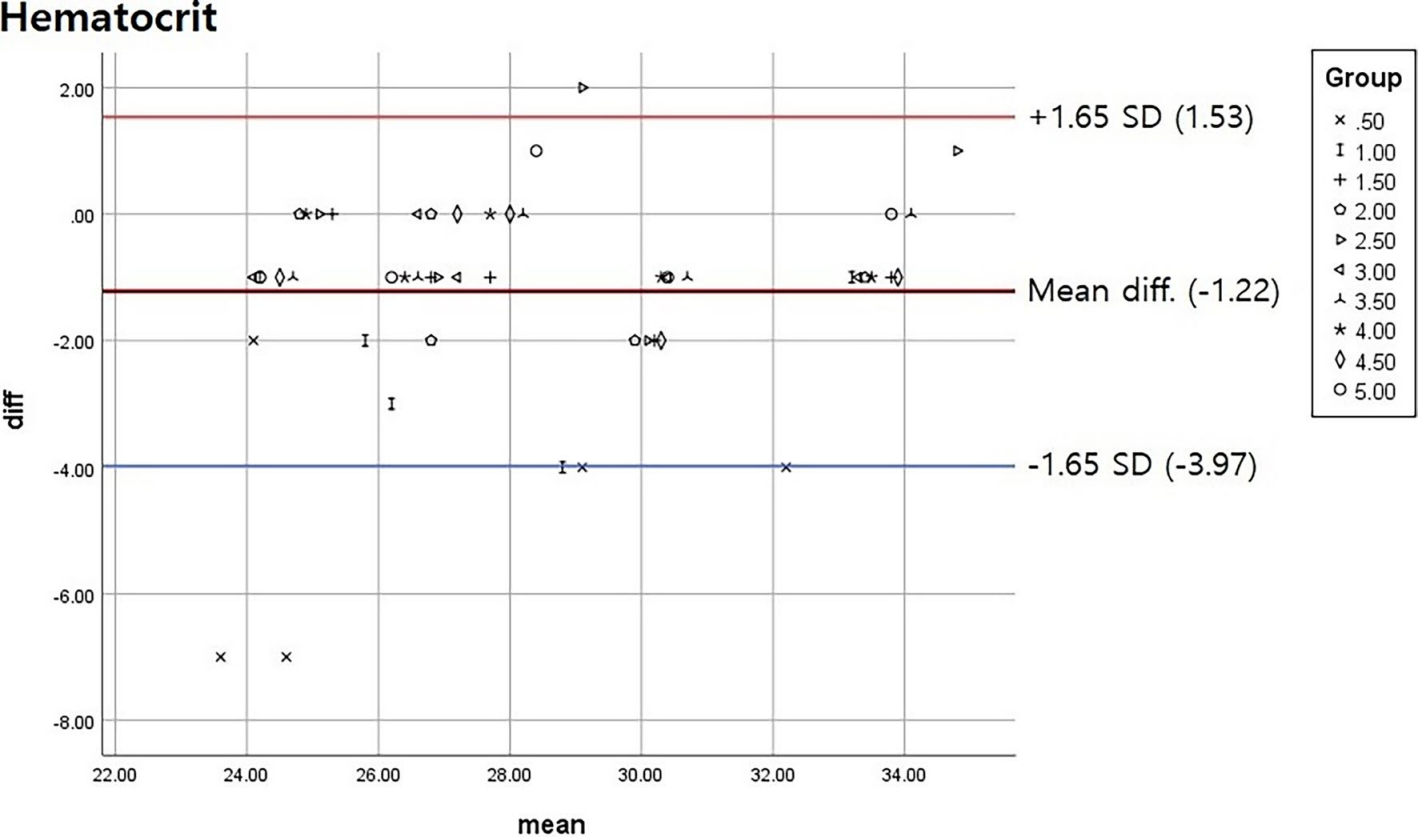
Hematocrit results for each clearing volume group. Results from all groups were within the 90% confidence interval after removal of the 2.5 mL group (Total n = 50). *SD* standard deviation, *diff* difference.

**Figure 3:**
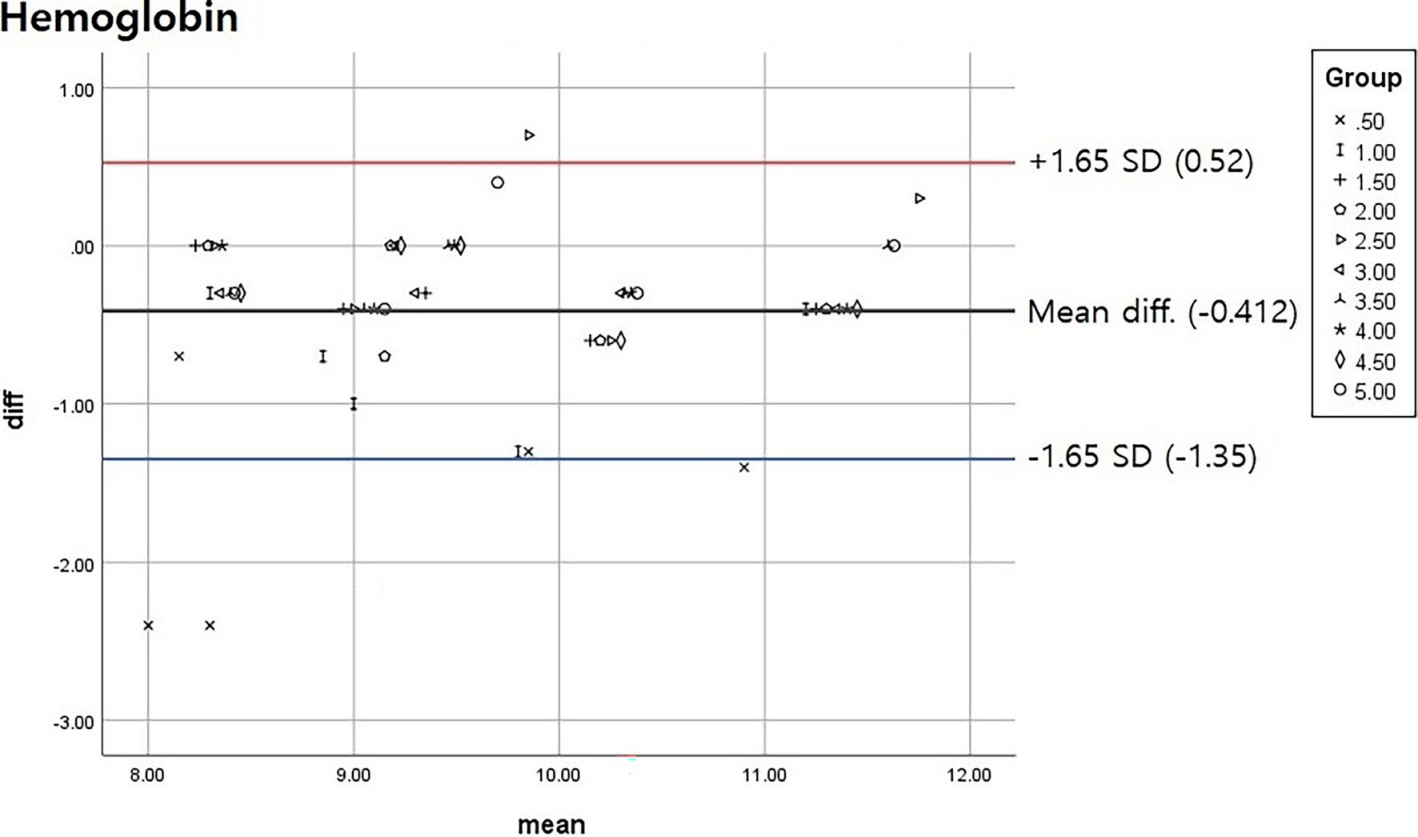
Hemoglobin results for each clearing volume group. Some results from the 0.5 and 1 mL clearing groups were just within the 90% confidence interval (Total n = 50). *SD* standard deviation, *diff* difference.

Our results showed that a clearing volume of 0.5 mL was insufficient for obtaining accurate blood test results, and removal of 1 mL gave borderline results. A single result from the 2.5 mL removal group was out of range, and all other groups were included in the 90% confidence interval (Fig. 2). The results for hemoglobin were similar to those for hematocrit (Fig. 3).

We analyzed the intra-class correlation coefficients by comparing the blood test results from the control and experimental HAMEL groups. All test results had significant intra-class correlation coefficients with p-values < 0.001, except for the results for base excess, chloride, and anion gap (Table 1).

**Table 1:** Intraclass correlation coefficient (ICC) results comparing the control and experimental groups.

	ICC (2,1)	95% CI	p-value
pH	0.990	0.982–0.994	< 0.001
pCO_2_	0.984	0.971–0.991	< 0.001
pO_2_	0.987	0.977–0.992	< 0.001
Base excess	0.890	0.806–0.938	< 0.001
Bicarbonate	0.928	0.874–0.959	< 0.001
Total CO_2_	0.912	0.846–0.950	< 0.001
Lactate	0.998	0.995–0.999	< 0.001
Sodium	0.913	0.846–0.950	< 0.001
Potassium	0.986	0.975–0.992	< 0.001
Chloride	0.648	0.380–0.800	< 0.001
Ionized calcium	0.988	0.979–0.993	< 0.001
Glucose	0.993	0.988–0.996	< 0.001
BUN	0.997	0.994–0.998	< 0.001
Creatinine	0.988	0.978–0.994	< 0.001
Hematocrit	0.965	0.939–0.980	< 0.001
Hemoglobin	0.955	0.922–0.975	< 0.001
Anion gap	0.719	0.506–0.840	< 0.001

Most results show ICC values higher than 0.9 with statistically significant p-values. The ICC model was a two-way random effect and single-measure model with absolute agreement.

*ICC* intra-class correlation coefficient, *pCO*_*2*_ partial pressure of carbon dioxide, *pO*_*2*_ partial pressure of oxygen, *CI* confidence interval, *BUN* blood urea nitrogen.

**Figure 4:**
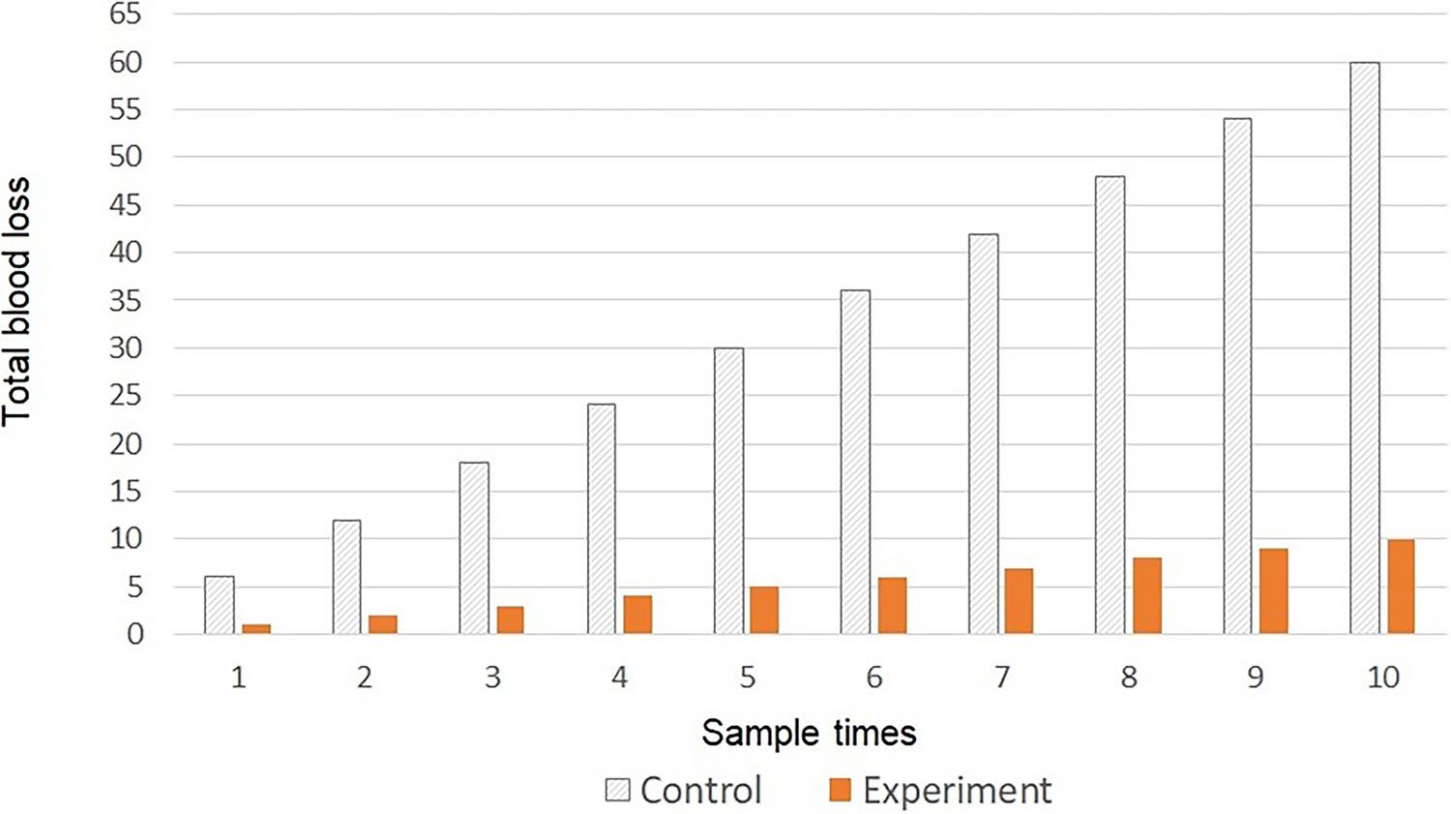
Difference in blood loss between the control and experimental groups. In the control group, 5 mL of dead space volume and 1 mL of sample volume were removed. In the experimental group, only 1 mL of sample volume was retrieved from the participant. (n = 50 for each group).

The total unnecessary blood loss from the control group was 5 mL per sample or 50 mL for 10 samples (Fig. 4), compared to 0 ml in the experimental group.

## Discussion

In our study, for reliable bleed test results, at least 3 mL of blood should be cleared prior to obtaining blood samples. Comparing blood tests which were sampled via traditional method and HAMEL proved that the test results are reliable. And as expected, HAMEL dramatically reduced unnecessary blood loss.

Previous studies have shown inconsistent results regarding the required A-line clearing volumes. Some studies have suggested a volume that is twice the dead space^1^, while others have presented absolute values such as 4 mL^2^. Moreover, additional removal is necessary if the normal saline used for flushing is heparinized^3^. Our results showed that 1.5 mL of dead space required a clearing volume of at least 3 mL prior to sampling for blood tests. Although this study was designed using a dead space of 1.5 mL and a total line length of approximately 33 cm, these characteristics can vary according to the A-line insertion site or individual ICU settings. To calibrate these differences, additional studies should be considered, and fluid lines should preferably be manufactured to simplify the calculation of the dead space by a simple length check.

Unfortunately, in the first sub-experiment, all ten blood samples of experimental group were compared with only one sample of control group, which was taken simultaneously with control group sample of 0.5 mL clearing volume. Due to the time gap between blood samples of 0.5 mL and 5 mL clearing volume, there could be distortion in the result which related to physiologic changes of pig.

According to Cicchetti et al.^11^, most of the intra-class correlation coefficient results were above 0.75, categorized as “Excellent”. Base excess result was below 0.9, but still reliable as it is classified as “Excellent”. Results of chloride and anion gap were in range of “Good” (0.6 to 0.75), 0.648 and 0.719 respectively. Since anion gap is affected by chloride level, we concluded that comparing serum chloride between two groups is main point for discussion. However, we could not identify proper reason for relatively low reliability of chloride level. Other electrolytes (Sodium, potassium, ionized calcium) presented excellent correlations, so dilution due to normal saline was not suspected. Since it was animal study, maybe we can find some other explanation when we perform clinical trials.

As the HAMEL system is feasible for A-line sampling, we expect that it will reduce blood loss, followed by fewer cases of anemia. This translates to a reduction in preventable transfusions and associated complications, which can ultimately reduce morbidity and mortality rates. Although blood transfusion is critical for some patients, it is associated with various complications^8,9^. The HAMEL system also minimizes manual manipulation of the A-line valve and valve orifice for syringe insertion; therefore, it may reduce blood loss due to human error and infectious environmental exposure.

With a fully returned clearing volume to the pig, the experimental group experienced much less blood loss than the control group. Even with the addition of a 1 mL sample volume, the control group showed dramatic blood loss compared to the experimental group. As many studies have reported the occurrence of iatrogenic anemia^4–7^, we expect the use of HAMEL to achieve better management of patient blood sampling.

HAMEL is not the world’s first system for closed control of clearing volume or blood preservation; Edward Inc. developed a similar system called the VAMP™ system. However, it had some issues that led to a recall, according to the United States Food and Drug Administration and Edward Inc. The VAMP™ system included a reservoir with a clearing volume of 5 mL; however, this large contact surface area created a risk of blood clot formation. Additionally, there was an iatrogenic risk of damping on the arterial blood pressure monitor when the stopcock and reservoir was malpositioned.

In this study as well, clotting was found to be an issue. This was mainly due to either the prolonged time during which the fluid line was filled with blood at sampling, which can cause sedimentation and blood clotting, or remnant blood inside the stopcock valve, which ultimately diffused through the fluid-line lumen to form a linear blood clot. In our study design, any remaining blood could be flushed out when the flushing volume was > 8 mL; however, this created the possibility of unnecessary fluid supplementation. Our team is considering an additional fluid line for maintaining the circulation of cleared-out blood during sampling to reduce the stationary time of blood flow.

Currently, blood sampling is still performed manually, and with each sample, the A-line is exposed to the external environment. To overcome these issues, we are considering automation of the A-line valve. Moreover, if we can connect this system to a point-of-care testing device, a fully automated bedside blood test may be possible for patients in the ICU.

## Conclusions

Our results showed that at least 3 mL of blood should be cleared before sampling for blood tests when using HAMEL.

Utilization of the HAMEL system did not show a bias in the blood test results compared with the control group. Based on reliable intra-class correlation coefficient values, the HAMEL system is not inferior to the traditional hand-sampling method.

In addition, sampling with the HAMEL system did not result in a loss of blood other than that used for blood tests; therefore, this system can reduce unnecessary blood loss.

## Data Availability

The datasets used and/or analyzed during the current study are available from the corresponding author upon reasonable request.

## Abbreviations

ICU: Intensive care unit
A-line: Arterial line
ICC: Intra-class correlation coefficient
pCO_2_: Partial pressure of carbon dioxide
pO_2_: Partial pressure of oxygen
CI: Confidence interval
BUN: Blood urea nitrogen
